# Plastic Forming of Sandwich Panels and Numerical Analyses of the Forming Processes Based on Elastoplastic Equivalent Model

**DOI:** 10.3390/ma14174955

**Published:** 2021-08-30

**Authors:** Ya Zhang, Qingmin Chen, Mingwei Wang, Xi Zhang, Zhongyi Cai

**Affiliations:** 1 College of Materials Science and Engineering, Jilin University, Changchun 130025, China; zhangya_216@163.com (Y.Z.); mwwang19@163.com (M.W.); zhan9x1@126.com (X.Z.); 2 Roll Forging Research Institute, Jilin University, Changchun 130025, China

**Keywords:** sandwich panels, plastic forming, equivalent model, orthotropy

## Abstract

This paper studies the plastic forming of sandwich panels and proposes a universal elastoplastic equivalent method suitable for sandwich panels. To verify the generality of the equivalent method, according to the different core structures, the cores of bi-directional trapezoidal sandwich (BTS) panels and aluminum foam sandwich (AFS) panels are equated to orthotropic and isotropic (special orthotropic) single-layer panels respectively. Through the finite element (FE) numerical simulation of the mesoscopic model of the sandwich panel, the elastoplastic constitutive relationship of the equivalent core model is established, and then the macroscopic equivalent model of the sandwich panel is established. The FE numerical simulation of plastic forming was carried out for the mesoscopic model and equivalent model of BTS panel and AFS panel, and plastic forming experiments were conducted for the sandwich panel through a multi-point forming (MPF) test machine. The results show that the relative errors of the section average stress at the same position of the equivalent model and the mesoscopic model of sandwich panels are all within 4%; compared with the experimental results, the equivalent model of the sandwich panel has high forming accuracy and small shape error, which verifies the high accuracy and generality of the equivalent method. Moreover, using the sandwich panel equivalent model effectively reduces the calculation time of the numerical simulation.

## 1. Introduction

Sandwich panels composed of high-strength and thin upper and lower face sheets and low-density core layers are widely used in aerospace, automotive industry, and construction due to their high strength to weight ratio and high energy absorption features [1]. For the needs of most engineering fields, sandwich panels are often formed into various complex shapes. In order to reduce costs and improve forming efficiency, a plastic forming process that directly forms the flat sandwich panel into the desired shape came into being. Cai et al. [2,3,4] conducted multi-point forming (MPF) experiments and finite element (FE) analysis on the egg-box-like sandwich panel to study the forming performance of the sandwich panel in the plastic forming process, where they obtained the conclusion that the forming performance of the egg-box-like sandwich panel is mainly affected by the defect model occurred in the plastic forming process and geometric parameters of sandwich panels. Subsequently, Cai et al. [5] successfully formed a defect-free double-curvature eggshell sandwich panel with positive and negative Gaussian curvature through MPF experiments. Zhang et al. [6,7] constructed a three-dimensional tetrakaidekahedral model, cubic-spherical, and three-dimensional Voronoi model to study the plastic forming of double-curvature aluminum foam sandwich panels.

Due to their discrete local geometry, it is difficult to model the core structures using FE methods directly, and the numerical simulation of the mesoscopic FE model of sandwich panels will generate huge time and economic costs [8]. Therefore, the core layer of the sandwich panel is usually homogenized to a single-layer continuum for easy analysis [9]. In the early studies, many scholars use the analytical method to study equivalent models of sandwich panels, such as the material mechanics method, the elastic mechanics method, and the energy method [10,11,12,13]. Rakesh et al. [14] obtained the constitutive equation of the equivalent sandwich model of honeycomb panels by using the strain energy method. Wang et al. [15] used the orthotropic constitutive model of the composite honeycomb core layer to establish a two-dimensional equivalent model to predict the natural frequency and mode shapes of sandwich panels. Wang et al. [16] conducted the modal test of the solar array panel to compare the honeycomb sandwich panel theory model, honeycomb panel theory model, and equivalent panel theory model, and the results show that the sandwich panel theory model has the highest equivalent accuracy. Liang et al. [17] clarified the bending forming performance and forming defects of a bi-directional trapezoidal sandwich panel in detail through MPF experiment, numerical simulation, and analytical methods.

However, the analytical process is complicated, and the analytical method is limited to the same type of sandwich panels. Fortunately, people found that combining FE technology with analytical methods can easily obtain the relevant parameters of the equivalent model of sandwich panels. Feng et al. [18] obtained the macroscopic equivalent elastic constants of the equivalent sandwich model through the FE analysis method, which was established by homogenizing the sandwich panel to a continuous single-layer panel. Then, the bending response of the actual and equivalent model of three typical sandwich panels was compared to verify the effectiveness of the equivalent model. Yuan et al. [19] proposed an equivalent modeling method in which the honeycomb core layer is equivalent to an orthotropic single-layer continuum and the upper and lower face sheets adopt shell elements. They used the method to obtain the elastic constitutive equations of the equivalent model by FE numerical simulation. Jiang et al. [20] raise a method to determine the equivalent elastic modulus of the core layer with experimental modal data and verified the accuracy of the method by FE analysis. In some practical engineering problems, the nonlinearity of the material constitutive relationship must be considered during analysis when a relatively large strain is formed under complex stress states. Considering this situation, Cui et al. [21] put forward an equivalent method to determine the equivalent nonlinear constitutive relationship of the honeycomb core layer on the basis of FE analysis on the mesoscopic structure of the honeycomb core layer after analyzing the nonlinear material problems of the honeycomb sandwich shells. Yet, the limitation of this equivalent method is obvious because it is only suitable for honeycomb sandwich panels.

Up to now, there are few studies on plastic forming with equivalent models of sandwich panels. Therefore, according to Hill’48 orthotropic yield criterion [22] and Hill anisotropic yield in the ABAQUS help documentation, this paper proposes a universal elastoplastic equivalent method of the sandwich panel where homogenizing the core layer into a single-layer continuum by the FE analysis of the mesoscopic structure of the core layer of the bi-directional trapezoidal sandwich (BTS) panel and the aluminum foam sandwich (AFS) panel, and the accuracy and generality of the equivalent model established through this method in the plastic forming process is verified.

## 2. Equivalent Model for Sandwich Panel

### 2.1. Bi-Directional Trapezoidal Sandwich Structure and Aluminum Foam Sandwich Structure

**Figure 1:**
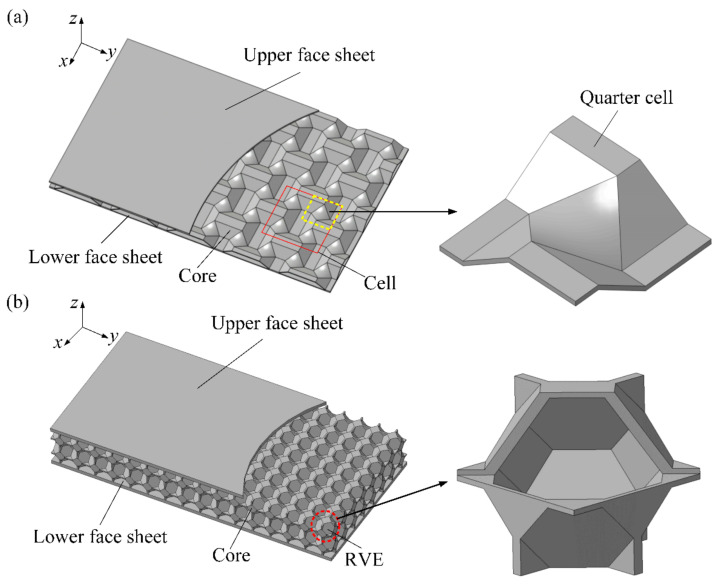
The structure of a sandwich panel. (**a**) Bi-directional trapezoidal sandwich panel (BTS panel); (**b**) aluminum foam sandwich panel (AFS panel).

The bi-directional trapezoidal sandwich (BTS) panel consists of upper and lower face sheets and a bi-directional trapezoidal core layer, as shown in Figure 1a. The core cell has a symmetrical structure, and a quarter of the core cell is composed of two convex and two concave platforms. The convex and concave platforms are connected by a trapezoidal oblique plane, and the inclined planes are transitioned by a quadrilateral curved surface. The size of a core cell is 47 mm × 47 mm × 6.5 mm.

Due to the disorder and complexity of the real core structure of the aluminum foam sandwich (AFS) panel, it takes a lot of time and energy to build a finite element (FE) model, whose meshing and calculation are also very difficult. In order to study the plastic forming of AFS panels, Zhang et al. [6] constructed a three-dimensional tetrakaidekahedral (TKD) model on the mesoscale which has periodic characteristics and proved to be an effective substitute for AFS panels. Therefore, this paper uses the TKD model to construct the core layer of the AFS panel, which is shown in Figure 1b. The mesoscopic representative volume element (RVE) model of the TKD model consists of six square faces, eight hexagonal faces, and twelve isosceles triangular faces. The size of the mesoscopic RVE is 5 mm × 5 mm × 5 mm.

### 2.2. Equivalent Core Elastic Parameters Calculation

In this section, the mesoscopic models of the core layers of the BTS panel and the AFS panel are established. FE analysis of the mesoscopic core model was performed by commercial software ABAQUS, and the elastic parameters of the equivalent core model with the same dimensions as the mesoscopic core model were obtained.

The core layer of the sandwich panel can be equated to an orthotropic homogeneous material. Based on the research of Robert [23] on the elastic constitutive relationship of orthotropic materials and the knowledge of elasticity, nine elastic parameters such as elastic modulus, shear modulus, and Poisson’s ratio are needed to establish the elastic constitutive relation of the equivalent core model. According to the generalized Hooke’s law, the elastic and shear modulus of the equivalent core model are obtained by uniaxial tensile and pure shear numerical simulations in the elastic deformation range of the mesoscopic core model, respectively.

**Figure 2:**
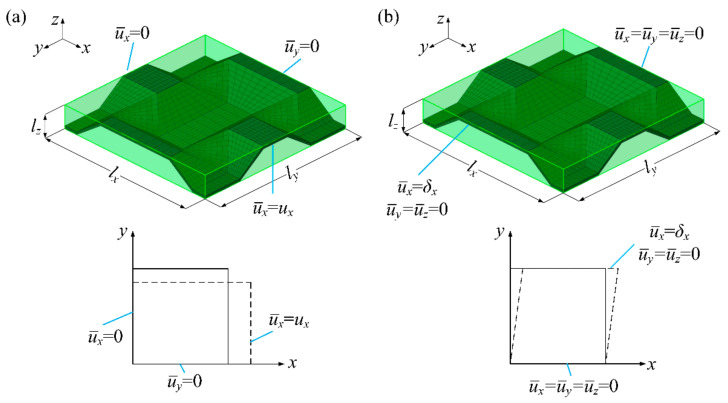
Boundary conditions. (**a**) Boundary conditions for $\bar{E}x$ (**b**) boundary conditions for $\bar{G}xy$.

Take the example of calculating the elastic modulus ${\bar{E}}_{x}$ and shear modulus ${\bar{G}}_{xy}$ for the equivalent core model of the BTS panel. To ensure the simulation accuracy, for uniaxial tension numerical simulation, the paper takes 21 RVEs in the length direction of the tension model and three RVEs in the width direction; for shear numerical simulation, six RVEs were taken in both the length and width directions. In order to easily observe and analyze, only one RVE is shown in the Figure 2 to describe the boundary conditions of the uniaxial tension and shear simulation. For uniaxial tensile and shear numerical simulations, the plane subjected to displacement constraints is coupled to a reference point by Equation, and a tensile or shear force is applied to the reference point to cause tensile or shear deformation to occur. ${\bar{E}}_{x}$ and ${\bar{\mu }}_{xy}$ of the equivalent core model can be obtained from Equations (1) and (2) respectively; ${\bar{G}}_{xy}$ can be acquired by Equation (3). ${\bar{E}}_{y}$, ${\bar{E}}_{z}$, ${\bar{\mu }}_{xz}$, ${\bar{\mu }}_{yz}$, ${\bar{G}}_{xz}$ and ${\bar{G}}_{yz}$ can also be obtained using the equations similar to Equations (1)–(3) by uniaxial tension and shear numerical simulation of the mesoscopic core structure, where *x*, *y*, *z* represent the elastic principal axes of the mesoscopic core model.

(1) $${\bar{E}}_{x}=\frac{{\bar{F}}_{x}^{\left(e\right)}l_{x}}{l_{y}l_{z}u_{x}}$$

(2) $${\bar{\mu }}_{xy}=-\frac{u_{y}l_{x}}{l_{y}u_{x}}$$

(3) $${\bar{G}}_{xy}=\frac{{\bar{T}}_{x}^{\left(e\right)}l_{y}}{l_{x}l_{z}\delta_{x}}$$

where the tension force ${\bar{F}}_{x}^{\left(e\right)}$ is imposed on the equivalent core model, and maintains the deformation of the tensile model within the elastic range and makes it produce displacement $u_{x}$ in the *x*-axis direction and $u_{y}$ in the *y*-axis direction, as Figure 2a shows; the shear force ${\bar{T}}_{x}^{\left(e\right)}$ is imposed on the equivalent core model, and maintains the deformation of the shear model within the elastic range and causes a certain displacement $\delta_{x}$ in the *x*-axis direction, where the superscript *e* indicates that the equivalent core model is in an elastic state under the action of the force.

The density of the equivalent core model is obtained by Equation (4):

(4) $$\bar{\rho }=\frac{V}{\bar{V}}\rho$$

where $\bar{\rho }$, $\bar{V}\;$ and ρ, V are the density and volume of the equivalent core model and the mesoscopic core model, respectively.

The elastic parameters of the equivalent core model of the BTS panel and AFS panel are given in Table 1.

### 2.3. Calculation of Anisotropic Parameters of Hill’48 Yield Criterion

The Hill’48 yield criterion is widely used in the sheet metal forming industry due to its simple structure and clear 3D expression as a quadratic function [24,25,26]. The general form of the yield criterion for the equivalent core model which is regarded as an orthotropic single-layer panel in the article by applying Hill’48 quadratic function is expressed as follows:

(5) $$2f(\sigma_{ij})=\bar{F}{\left(\sigma_{y}-\sigma_{z}\right)}^{2}+\bar{G}{\left(\sigma_{z}-\sigma_{x}\right)}^{2}+\bar{H}{\left(\sigma_{x}-\sigma_{y}\right)}^{2}+2\bar{L}\tau_{yz}{}^{2}+2\bar{M}\tau_{zx}{}^{2}+2\bar{N}\tau_{xy}{}^{2}=1$$

where *x*, *y*, *z* represent the orthogonal principal axes of the equivalent core model, $\bar{F}$, $\bar{G}$, $\bar{H}$, $\bar{L}$, $\bar{M}$ and $\bar{N}$ are the anisotropic parameters obtained by numerical simulations of the mesoscopic core model in different orientations, and $\sigma_{x}$, $\sigma_{y}$, $\sigma_{z}$, $\tau_{xy}$, $\tau_{yz}$ and $\tau_{zx}$ are the stress components at any point in the equivalent core model.

For the equivalent core model, the anisotropic parameters can be obtained from Equation (6):

(6) $$\left\{\begin{array}{c} \bar{F}=\frac{{\bar{\sigma }}_{sx}^{2}}{2}\left(\frac{1}{{\bar{\sigma }}_{sy}^{2}}+\frac{1}{{\bar{\sigma }}_{sz}^{2}}-\frac{1}{{\bar{\sigma }}_{sx}^{2}}\right)\\ \bar{G}=\frac{{\bar{\sigma }}_{sx}^{2}}{2}\left(\frac{1}{{\bar{\sigma }}_{sz}^{2}}+\frac{1}{{\bar{\sigma }}_{sx}^{2}}-\frac{1}{{\bar{\sigma }}_{sy}^{2}}\right)\\ \bar{H}=\frac{{\bar{\sigma }}_{sx}^{2}}{2}\left(\frac{1}{{\bar{\sigma }}_{sx}^{2}}+\frac{1}{{\bar{\sigma }}_{sy}^{2}}-\frac{1}{{\bar{\sigma }}_{sz}^{2}}\right)\\ \bar{L}=\frac{1}{2}{\left(\frac{{\bar{\sigma }}_{sx}}{{\bar{\sigma }}_{syz}}\right)}^{2}\\ \bar{M}=\frac{1}{2}{\left(\frac{{\bar{\sigma }}_{sx}}{{\bar{\sigma }}_{sxz}}\right)}^{2}\\ \bar{N}=\frac{1}{2}{\left(\frac{{\bar{\sigma }}_{sx}}{{\bar{\sigma }}_{sxy}}\right)}^{2} \end{array}\right.$$

where ${\bar{\sigma }}_{sx}$, ${\bar{\sigma }}_{sy}$, ${\bar{\sigma }}_{sz}$, ${\bar{\sigma }}_{sxy}$, ${\bar{\sigma }}_{syz}$ and ${\bar{\sigma }}_{sxz}$ are the yield stresses of the equivalent core model when the FE model begins to yield and the subscript *s* indicates that the equivalent core model just begins to yield. These yield stresses are obtained by numerical simulations of uniaxial tension and shear for the mesoscopic core model, as shown in Figure 2.

**Figure 3:**
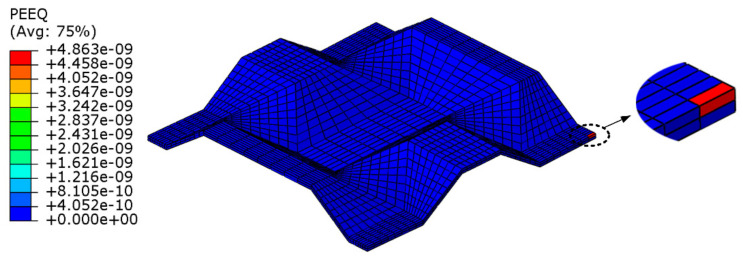
Initial yield model of the mesoscopic core model.

Taking the calculation of the yield stress ${\bar{\sigma }}_{sx}$ of the equivalent core model of the BTS panel as an example, the paper conducts uniaxial tensile numerical simulation on mesoscopic core model of the BTS panel. The equivalent plastic strain (PEEQ) of the mesoscopic core model is used as the basis for judging the beginning of yielding, as Figure 3 shows. When PEEQ is greater than 0, it indicates that mesoscopic core model just begins to yield.

The tension force ${\bar{F}}_{x}^{\left(p\right)}$ applied to the equivalent core model is extracted as the yield load when PEEQ of the mesoscopic core model is just greater than 0, where the superscript *p* indicates that the equivalent core model just begins to yield under the action of the force.

Then, ${\bar{\sigma }}_{sx}$ can be calculated by Equation (7). ${\bar{\sigma }}_{sy}$, ${\bar{\sigma }}_{sz}$, ${\bar{\sigma }}_{sxy}$, ${\bar{\sigma }}_{syz}$ and ${\bar{\sigma }}_{sxz}$ can also be obtained using the equations similar to Equation (7) through numerical simulation for the mesoscopic core model.

(7) $${\bar{\sigma }}_{sx}=\frac{{\bar{F}}_{x}^{\left(p\right)}}{l_{y}l_{z}}$$

The calculation results of anisotropic parameters of the equivalent core model of the BTS panel and AFS panel are summarized in Table 2.

### 2.4. Equivalent Stress-Strain Curve of Equivalent Core Model

The equivalent stress for the equivalent core model can be defined as:

(8) $$\bar{\sigma }=\sqrt{\bar{F}{\left(\sigma_{y}-\sigma_{z}\right)}^{2}+\bar{G}{\left(\sigma_{z}-\sigma_{x}\right)}^{2}+\bar{H}{\left(\sigma_{x}-\sigma_{y}\right)}^{2}+2\bar{L}\tau_{yz}^{2}+2\bar{M}\tau_{zx}^{2}+2\bar{N}\tau_{xy}^{2}}$$

By applying the law of flow, the expression of the equivalent plastic strain increment can be derived:

(9) $$d{\bar{\varepsilon }}_{p}=\sqrt{\frac{\bar{F}{\left(\bar{G}d\varepsilon_{y}^{p}-\bar{H}d\varepsilon_{z}^{p}\right)}^{2}+\bar{G}{\left(\bar{H}d\varepsilon_{z}^{p}-\bar{F}d\varepsilon_{x}^{p}\right)}^{2}+\bar{H}{\left(\bar{F}d\varepsilon_{x}^{p}-\bar{G}d\varepsilon_{y}^{p}\right)}^{2}}{{\left(\bar{F}\bar{G}+\bar{G}\bar{H}+\bar{H}\bar{F}\right)}^{2}}+\frac{2d\varepsilon_{yz}^{p}{}^{2}}{\bar{L}}+\frac{2d\varepsilon_{zx}^{p}{}^{2}}{\bar{M}}+\frac{2d\varepsilon_{xy}^{p}{}^{2}}{\bar{N}}}$$

Combining Equation (6), the relationship between the stress (σ) and plastic strain increment ($d\varepsilon_{p}$) of mesoscopic core model which are obtained by numerical simulation of uniaxial tension along the *x*-direction and equivalent stress and equivalent plastic strain increment of the equivalent core model becomes:

(10) $$\left\{\begin{array}{c} \bar{\sigma }=\sigma \\ d{\bar{\varepsilon }}_{p}=d\varepsilon_{p} \end{array}\right.$$

Due to the advantages of a simple and accurate integration process, the paper uses a classical forward Euler algorithm for stress update, which includes elastic prediction and plastic correction [27].

The elastic prediction can be obtained from the elasticity equation of the total strain increment (the increments at the current step *n*+1 are taken from previous step *n*):

(11) $$\sigma_{n+1}^{tri}=\sigma_{n}+D^{e}\cdot d\varepsilon_{n+1}$$

where $D^{e}$, $\sigma^{tri}$ and dε are the stiffness matrix of the equivalent core model, trial stress tensor, and strain increment tensor, respectively.

**Figure 4:**
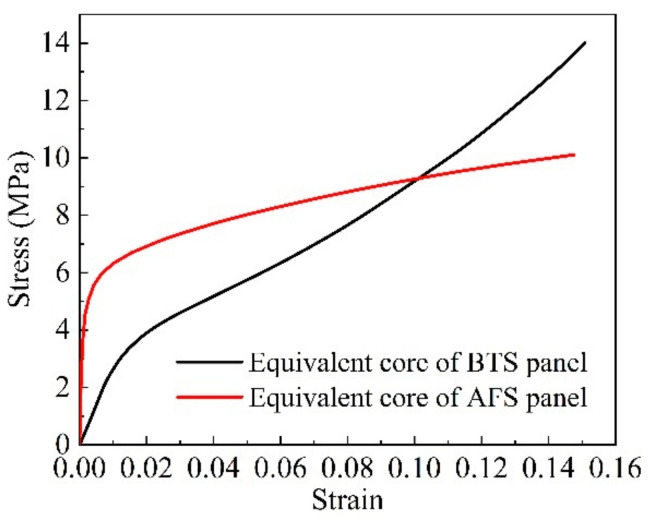
*x*-direction uniaxial tensile stress-strain curve of the equivalent core model.

If the trial stress exceeds the yield surface, the plastic corrector step is employed,

(12) $$d\lambda =\frac{\frac{\partial f}{\partial \sigma }:D^{e}:d\varepsilon }{\frac{\partial f}{\partial \sigma }:D^{e}:\frac{\partial f}{\partial \sigma }+\bar{H^{\prime }}}$$

(13) $$\bar{H^{\prime }}=\frac{{\bar{E}}_{x}E^{\prime }}{{\bar{E}}_{x}-E^{\prime }}$$

(14) $$\sigma_{n+1}=\sigma_{n+1}^{tri}-d\lambda D^{e}\frac{\partial f}{\partial \sigma }$$

where the isotropic strengthening plastic modulus ${\bar{H}}^{\prime }$ is the slope of the stress-strain curve shown in Figure 4 and $E^{\prime }=d\sigma /d\varepsilon$; f is the yield function of the equivalent core model in Equation (5); σ and dλ are modified stress tensor and plastic multiplier increment, respectively.

### 2.5. Numerical Simulation of MPF of Sandwich Panel FE Model

By analyzing and calculating the elastoplastic parameters of the equivalent core model of the sandwich panels, the macroscopic equivalent model of the sandwich panel is established. The numerical simulations of the MPF and springback process are performed for the mesoscopic model and equivalent model of BTS panels and AFS panels by commercial software ABAQUS (6.14), and three different shapes of sandwich panels were obtained: cylindrical, spherical, and saddle-shaped.

**Figure 5:**
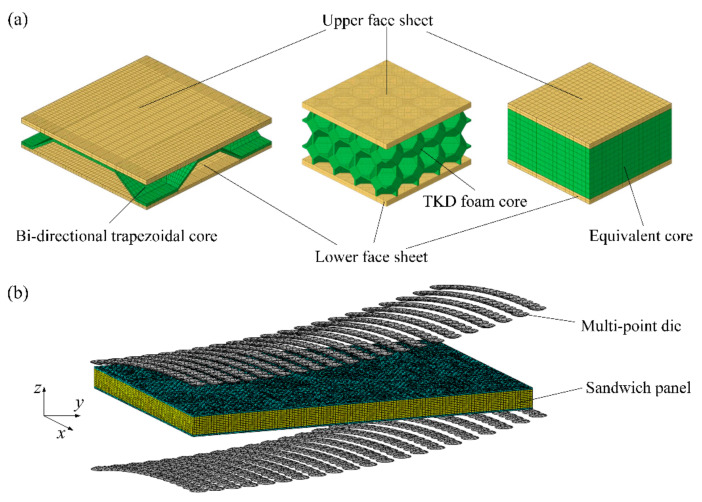
FE model. (**a**) FE model of sandwich panels; (**b**) MPF FE model of sandwich panel.

For BTS panels, the thickness of the upper and lower face sheets of the sandwich panel is 1.5 and 1.0 mm, respectively, and the height of the core is 6.5 mm. The dimension of the mesoscopic FE model for cylindrical forming is 329 mm × 94 mm × 9 mm, and the dimensions of the mesoscopic FE model for spherical and saddle-shaped forming are 517 mm × 376 mm × 9 mm. For AFS panels, the thickness of both upper and lower face sheets is 1.0 mm, and the height of the core is 10 mm. Using the symmetry of the sandwich panel, this paper takes a quarter of the sandwich panel to establish the mesoscopic FE model to reduce the computational cost. The dimension of the mesoscopic FE model for cylindrical forming is 150 mm × 50 mm × 12 mm, and the dimensions of the mesoscopic FE model for spherical and saddle-shaped forming are 200 mm × 150 mm × 12 mm. The detailed material parameters of the face sheet and core of the mesoscopic model of the BTS panel and AFS panel are listed in Table 3. For the sandwich panel equivalent model, the dimensions of the equivalent models of the two sandwich panels are consistent with the dimensions of their mesoscopic models, and the face sheet material of the sandwich panel equivalent model is consistent with the mesoscopic model, and the elastoplastic parameters of the equivalent core are obtained by FE analysis. As shown in Figure 5a, the C3D8R element was used for FE modeling of the face sheet and core of the BTS panel mesoscopic model, and C3D6 elements and C3D8R elements were used for FE modeling of the face sheet and core of the AFS panel mesoscopic model. According to the dimensions of the equivalent models of BTS panels and AFS panels, C3D8R elements with an element size of 2.35 mm were used to model the face sheet and core of the equivalent model of the BTS panel, and C3D8R elements with an element size of 2 mm were used to model the face sheet and core of the equivalent model of the AFS panel. The R3D4 element was used to model the multi-point die punch. A package of dynamic explicit and static general was carried out to simulate the bending and springback process, respectively. The contact type during the numerical simulation is general contact. Figure 5b shows the MPF FE model of the sandwich panel.

## 3. Analysis of Numerical Simulation Results

### 3.1. Simulation Results and Analysis of Sandwich Panel FE Model

**Figure 6:**
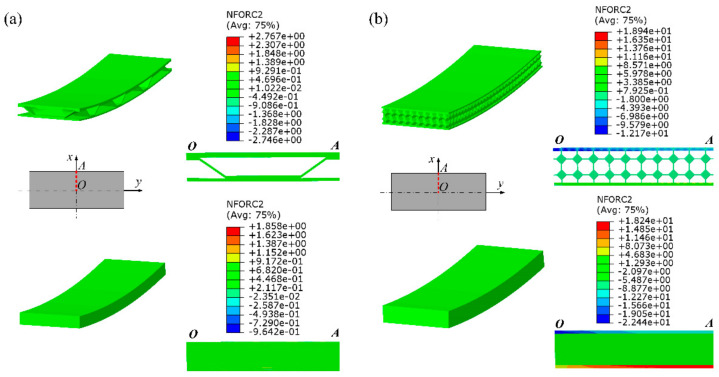
Nodal force distribution in the section of sandwich panel. (**a**) Mesoscopic model and equivalent model of BTS panel; (**b**) mesoscopic model and equivalent model of AFS panel.

In order to verify the validity of the elastoplastic equivalent model of the sandwich panel, this paper compares the average stress value of the characteristic sections at the same position of the mesoscopic model and the equivalent model. The section average stress value is calculated by dividing the sum of the absolute values of the nodal forces perpendicular to the section of all nodes in the section by the corresponding cross-sectional area. For cylindrical forming numerical simulations of the BTS panel and AFS panel with a forming radius of 600 mm, this paper investigates the average stress at the OA section position, and the average stress values of the OA section are obtained from the numerical simulation results of their mesoscopic models and equivalent models. Figure 6 shows the nodal force distribution at the OA section location.

**Figure 7:**
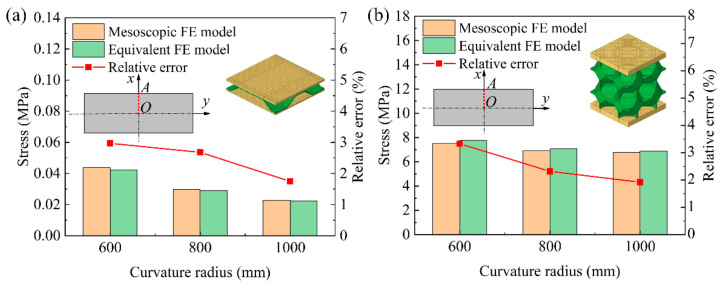
Section average stresses of the mesoscopic model and equivalent model of cylindrical sandwich panel. (**a**) BTS panel; (**b**) AFS panel.

Figure 7 shows the comparison results of the numerical simulation results of the average stress at the symmetrical section OA of the mesoscopic model and the equivalent model of the BTS panel and the AFS panel with different cylindrical forming radius. From Figure 7a, it can be seen that the average stress in the section of the equivalent model of the BTS panel is slightly lower than that of the mesoscopic model at the same forming radius. As the forming radius increases, the average stress in the section of the mesoscopic model and the equivalent model gradually decreases. The relative error of the section average stress of the equivalent model and the mesoscopic model is gradually reduced with the forming radius, and the maximum relative error and minimum relative error are 2.97% and 1.75%, respectively. It can be seen from Figure 7b that the section average stress of the mesoscopic model and the equivalent model of AFS panel and the relative errors of the section average stress vary with the forming radius in the same trend as that of the BTS panel, but the section average stress of the equivalent model of AFS panel is slightly larger than that of the mesoscopic model, and the maximum relative error and the minimum relative error are 3.33% and 1.92%, respectively. By comparing the average stresses in the section of the mesoscopic model and the equivalent model of the above two sandwich panels, it shows that the equivalent model of the sandwich panel has good accuracy under the cylindrical forming type.

**Figure 8:**
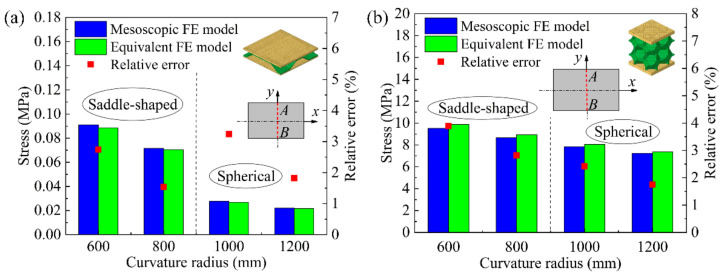
Section average stresses of the mesoscopic and equivalent models of the saddle-shaped and spherical sandwich panels. (**a**) BTS panel; (**b**) AFS panel.

Figure 8 shows the comparison results of numerical simulation of the average stress in the cross-section at the center AB of the long edge of the mesoscopic model and the equivalent model of the BTS panel and the AFS panel with different shapes. Figure 8a shows the comparison of the average stress in the cross-section of the mesoscopic model and the equivalent model of the saddle-shaped and spherical BTS panel. The relative error of the section average stress of the mesoscopic model and the equivalent model of the spherical BTS panel with a forming radius of 1000 mm is the largest, and the maximum relative error is 3.23%. Figure 8b shows the comparison of the average stress in the cross-section of the mesoscopic model and the equivalent model of the saddle-shaped and spherical AFS panel. As can be seen, the relative error of the section average stress of the mesoscopic model and the equivalent model of saddle-shaped AFS panel with a forming radius of 600 mm is the largest, and the maximum relative error is 3.74%. The result shows that the maximum relative error of the section average stress of both the mesoscopic model and the equivalent model of the two sandwich panels are less than 4%, which indicates that the elastoplastic parameters of the equivalent model of the sandwich panels calculated by the finite element analysis are highly accurate.

### 3.2. Effect of Element Size on Numerical Simulation Results

To further verify the accuracy of the equivalent models of BTS panels and AFS panels, numerical simulations of the equivalent models of sandwich panels with different element sizes are conducted in this paper. According to the dimension of the equivalent model of sandwich panel, the equivalent FE model of BTS panel is established with the element size of 1, 2.35, and 4.7 mm respectively, and the equivalent FE model of the AFS panel is established with the element size of 1, 2, and 2.5 mm, respectively.

**Figure 9:**
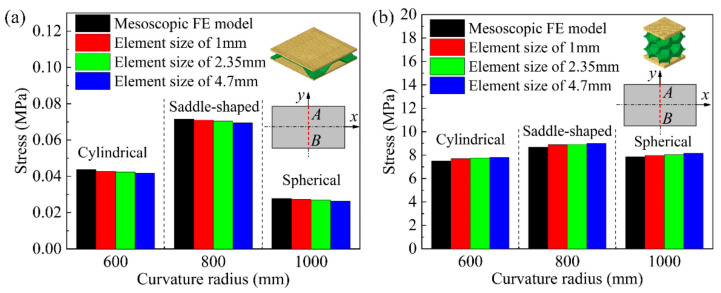
Section average stress of the mesoscopic model of the sandwich panel and the equivalent model of the sandwich panel with different element sizes. (**a**) BTS panel; (**b**) AFS panel.

Under different forming types, the average stresses in the cross-section of the equivalent model of the sandwich panel with different element sizes are compared with the mesoscopic model of the sandwich panel as shown in Figure 9. The extracted cross-section AB is located at the center of the long edge of the sandwich panel FE model. From Figure 9a,b, it can be seen that the change of the element size of the equivalent model of the sandwich panel has little effect on the results of the average stress in the cross-section, but overall, it shows that the smaller the element size, the closer the average stress in the cross-section between the equivalent model and the mesoscopic model.

In this paper, a large number of plastic-forming numerical simulations are carried out for the mesoscopic model and equivalent model of the BTS panel and the AFS panel. The CPU of the server used in the numerical simulation calculation is E5-2697 V2 and the RAM is 32 GB. In order to obtain the effect of element size on the calculation time of numerical simulation, this paper compares the calculation time of equivalent models of sandwich panels with different element sizes. The calculation time of numerical simulation of the sandwich panel mesoscopic model and the equivalent model of sandwich panels with different element sizes are listed in Table 4. It can be seen from Table 4 that the calculation time of the equivalent models for the BTS panel and the AFS panel is shorter compared to the calculation time of the mesoscopic model. Particularly in the numerical simulation calculation of the mesoscopic model and the equivalent model of the AFS panel, the equivalent model of the AFS panel can significantly reduce the numerical calculation time. By comparing the calculation time of numerical simulation of FE models of sandwich panels with different element sizes, it is found that the calculation time of numerical simulation of equivalent models of sandwich panels gradually increases as the element size decreases.

Combined with the analysis results in Figure 9 and Table 4, the equivalent model of the BTS panel established with an element size of 2.35 mm and the equivalent model of the AFS panel established with an element size of 2 mm can ensure the accuracy of the numerical simulation results and save the calculation time of numerical simulation effectively.

### 3.3. Forming Accuracy of Sandwich Panel Equivalent Model

Table 5 lists the comparison of the radius of the mesoscopic and equivalent models of the BTS panel and AFS panel after springback with different cylindrical bending radius. As can be seen from Table 5, the equivalent model of the BTS panel has a slightly smaller radius after springback than the mesoscopic model under the same cylindrical bending radius, and the maximum relative error of the radius after springback of the mesoscopic model and the equivalent model of BTS panels with a cylindrical bending radius of 600, 800, and 1000 mm is only 0.65%. Comparing the radius after springback of the mesoscopic model and the equivalent model of the AFS panel, the radius after springback of the equivalent model is slightly larger than that of the mesoscopic model under the same cylindrical bending radius, and the maximum relative error of the radius after springback of the equivalent model and the mesoscopic model is only 1.56%. By comparison, it is found that the radius after springback of the mesoscopic model and the equivalent model of the BTS panel and the AFS panel have no significant difference, which indicates that the equivalent model of the sandwich panel has high forming accuracy.

**Figure 10:**
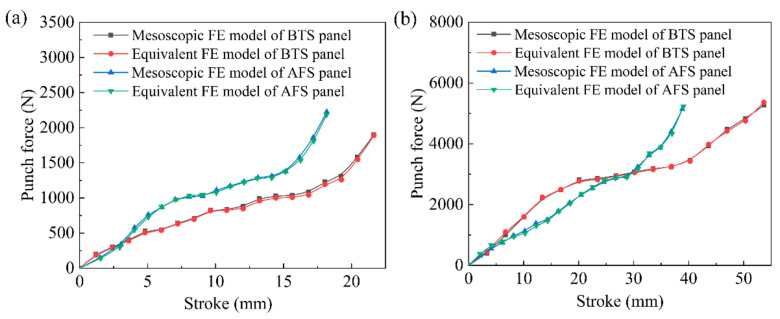
Load-stroke curves for the mesoscopic model and equivalent model of sandwich panels. (**a**) Cylindrical sandwich panels with a radius of 600 mm; (**b**) saddle-shaped sandwich panels with a radius of 800 mm.

Figure 10 illustrates the load-stroke curves of the mesoscopic model and the equivalent model of sandwich panels with different shapes. As can be seen in Figure 10a,b, the load-stroke curves of the mesoscopic model and the equivalent model of the BTS panel and the AFS panel with the same shape are basically the same, which proves that the equivalent model of the sandwich panel all have high forming accuracy with different forming types.

## 4. MPF Experiments and Verification

### 4.1. MPF Experiments of Sandwich Panels

**Figure 11:**
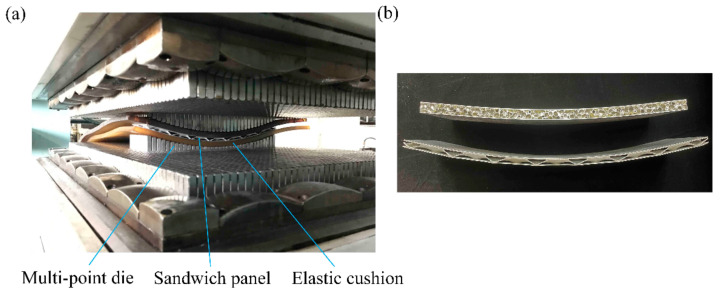
MPF experiment. (**a**) MPF process; (**b**) bending test experimental parts.

In order to obtain BTS panels and AFS panels with three different shapes: cylindrical, spherical, and saddle-shaped, this paper uses multi-point forming (MPF) technology to carry out plastic forming experiments on sandwich panels [28,29]. MPF technology is an advanced sheet forming technology, which replaces the traditional die with a regular array of punch points and forms a flexible die with variable shape by computer control of punch positions, thus realizing rapid sheet forming, which not only reduces the die manufacturing cost but also saves time. The multi-point die punch element is a hemisphere with a radius of 10 mm, and the face sheet and core materials of the BTS panel and the AFS panel are shown in Table 3. The dimensions of the sandwich panels used in the MPF experiments are the same as the dimensions of the mesoscopic FE models of the sandwich panels during the numerical simulation. Figure 11a shows the experimental process of MPF of sandwich panels.

**Figure 12:**
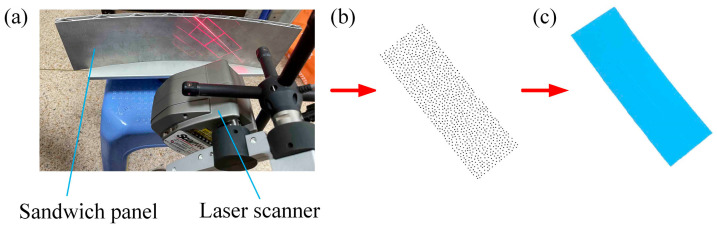
Surface reconstruction process of sandwich panels. (**a**) 3D scanning; (**b**) point cloud data; (**c**) reconstructed surface.

To verify the accuracy of the equivalent model of sandwich panels, the point cloud data of the BTS panel and AFS panel forming parts were obtained by using a 3D laser scanning method. The 3D scanner used in the experimental process is an NDI handheld 3D laser scanner (NDI, Waterloo, Canada) measurement accuracy of not more than 0.04 mm, which can scan the workpiece of 0–20 m. The point cloud data of numerical simulation parts and experimental parts were imported into the engineering software GEOMAGIC QUALIFY 2013, and by using the inverse analysis capability of the software, the corresponding surface shapes were reconstructed, and finally, the error analysis of the surface shapes of the experimental and simulated parts of the sandwich panel was carried out. Figure 12 shows the surface reconstruction process of sandwich panels.

### 4.2. Experimental Results and Analyses

The shape error is an important index to measure the accuracy of plastic forming of sandwich panels. This paper verifies that the elastoplastic equivalent method of the sandwich panel is well applicable to plastic forming by comparing the shape error of the FE model of the sandwich panel and experimental parts.

**Figure 13:**
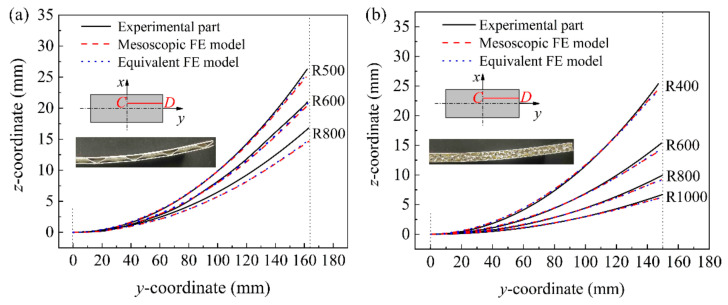
Longitudinal section of the upper face sheet of cylindrical sandwich panels with different forming radius after springback. (**a**) BTS panel; (**b**) AFS panel.

Figure 13 shows the comparison results of the longitudinal section of the upper face sheet after springback of the experimental and simulated parts of the BTS panel and AFS panel with different cylindrical-forming radius. Due to the symmetry of the sandwich panel, half of the sandwich panel in the long axis direction is taken as the research object. The location of the extracted profile is located at 20 mm from the center of the *y*-direction on the right half of the cylindrical bending direction. Figure 13a shows the comparison results of the cross-sectional profiles of the upper face sheet of the experimental and simulated BTS panel with a cylindrical forming radius of 500, 600, and 800 mm. It can be seen from Figure 13a that the cross-sectional profile of the equivalent model and the mesoscopic model of the BTS panel basically overlap, and the cross-sectional profile of the equivalent model and the experimental part overlap near the center, and the springback of the equivalent model of the sandwich panel is slightly larger than that of the experimental part at the edge position, thus the deviation of the cross-sectional profile becomes larger. Figure 13b shows the comparison results of the cross-sectional profile of the upper face sheet of the experimental and simulated parts of the AFS panel with a cylindrical forming radius of 400, 600, 800, and 1000 mm. As can be seen in Figure 13b, the upper face sheet cross-sectional profile of the equivalent model of the AFS panel overlaps highly with the mesoscopic model and the experimental parts. The result shows that the equivalent model of the sandwich panel has high forming accuracy.

**Figure 14:**
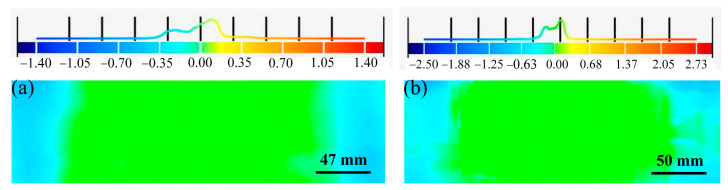
Shape errors on the upper face sheet of the sandwich panel equivalent model. (**a**) BTS panel; (**b**) AFS panel.

For the analysis of a cylindrical sandwich panel with a radius of 600 mm after springback, the shape errors of the upper face sheet of the equivalent model of the sandwich panel in the normal direction were measured by taking the upper face sheet of the experimental part as the reference surface, as displayed in Figure 14a,b. The shape errors of the equivalent models of BTS panels and AFS panels are −1.40 to 1.40 mm and −2.50 to 2.73 mm, respectively. All the results show that the shape errors of the equivalent models of the two sandwich panels are very small, indicating that the equivalent models of the sandwich panels have high forming accuracy.

## 5. Conclusions

This paper introduces a universal FE equivalent method applicable to the elastoplastic range of sandwich panels and establishes an equivalent model of sandwich panels. Numerical simulations and MPF experiments were performed to investigate the plastic forming process of BTS panels, AFS panels, and their equivalent models. The main conclusions are as follows:

- The core layers of the BTS panels and AFS panels were equated to an orthotropic single-layer panel, and the elastic parameters, anisotropy parameters, and equivalent stress-strain curves of the equivalent model of the sandwich panel were obtained by numerical simulations of uniaxial tension and pure shear of the mesoscopic model of the sandwich panel.
- Numerical simulations of cylindrical, spherical, and saddle-shaped forming were carried out for the mesoscopic and equivalent models of BTS panels and AFS panels. The average stresses in the cross-section of the mesoscopic and equivalent models of the same shaped sandwich panels at the same position were compared, and the maximum relative error of the average stresses in the cross-section did not exceed 4%.
- The effects of equivalent models of sandwich panels with different element sizes on numerical simulation results and calculation time are investigated. The results show that the equivalent model of the BTS panel established with an element size of 2.35 mm and the equivalent model of the AFS panel established with an element size of 2 mm can effectively reduce the calculation time of numerical simulations on the basis of ensuring accuracy.
- The longitudinal section of the upper face sheet of the equivalent model of the BTS panel and AFS panel fits well with the longitudinal section of the upper panel of the mesoscopic model and the experimental parts, and the shape error of the equivalent model of the sandwich panel is small compared with the experimental parts, which indicates that the equivalent models of BTS panels and AFS panels have good forming accuracy and verifies the high accuracy and generality of the equivalent method.

## Figures and Tables

**Table 1:** Elastic parameters of the equivalent core model.

Core Type	$\bar{\rho }$ /(kg/m^3^)	${\bar{E}}_{x}$ /MPa	${\bar{E}}_{y}$ /MPa	${\bar{E}}_{z}$ /MPa	${\bar{\mu }}_{xy}$	${\bar{\mu }}_{xz}$	${\bar{\mu }}_{yz}$	${\bar{G}}_{xy}$ /MPa	${\bar{G}}_{xz}$ /MPa	${\bar{G}}_{yz}$ /MPa
BTS	335	670	613	66	0.23	0.26	0.46	120	738	787
AFS	563	4629	4629	4629	0.17	0.17	0.17	572	572	572

**Table 2:** Anisotropic parameters of the equivalent core model.

Core Type	$\bar{F}$	$\bar{G}$	$\bar{H}$	$\bar{L}$	$\bar{M}$	$\bar{N}$
BTS	0.875	1.449	−0.449	0.768	1.305	5.547
AFS	0.5	0.5	0.5	15.208	15.208	15.208

**Table 3:** Material constants of the sandwich panel.

Type	Structure	Young’s Modulus (MPa)	Yield Stress (MPa)	Poisson’s Ratio	Density (kg/m^3^)
BTS panel	Face sheet	69,000	45	0.33	2800
	Core	71,000	128	0.33	2720
AFS panel	Face sheet	70,300	193	0.33	2680
	Core	63,000	63	0.33	2700

**Table 4:** Calculation time for numerical simulation of FE model of the sandwich panel.

Forming Type	Forming Radius (mm)	BTS Panel				AFS Panel			
		Mesoscopic FE Model Calculation Time (h)	Equivalent FE Model Calculation Time (h)			Mesoscopic FE Model Calculation Time (h)	Equivalent FE Model Calculation Time (h)		
			Element Size (mm)				Element Size (mm)		
			1	2.35	4.7		1	2	2.5
Cylindrical	600	1.60	0.52	0.40	0.32	16.53	1.54	1.28	1.25
Saddle-shaped	800	6.18	2.05	1.72	1.60	59.07	2.07	1.73	1.68
Spherical	1000	5.70	1.93	1.65	1.54	58.02	1.84	1.53	1.48

**Table 5:** Radius comparison after springback of the sandwich panel.

Type	Forming Radius (mm)	Mesoscopic FE Model (mm)	Equivalent FE Model (mm)	Relative Error (%)
BTS panel	600	649.15	644.90	0.65
	800	901.85	898.03	0.42
	1000	1168.31	1169.30	0.08
AFS panel	600	775.83	779.83	0.52
	800	1199.23	1214.30	1.26
	1000	1774.65	1802.36	1.56

## Data Availability

Not applicable.
